# Mr. Dunning, on Inoculation

**Published:** 1805-10-01

**Authors:** Richard Dunning

**Affiliations:** Dock


### *To the Editors of the Medical and Physical Journal.*

GENTLEMEN,

ON Monday, the 29th of July last, a child of Mr. Jackman, joiner, in Clowance Street, two years and a half old, was attacked with the usual symptoms of fever, but not violently, for the child played much of this day. The day before this child was in every respect perfectly well, and was taken several miles into the country. On Tuesday several eruptions were observed, and on Wednesday and Thursday these became numerous, amounting to many hundreds, and were principally on the face and extremities. On Wednesday a few of them were surrounded with that remarkable efflorescence which we have here often noticed, and which is so accurately described and insisted on by Messrs. Little and Smith in their respective letters addressed to me, and published in No. 77 of your Journal. Mr. Little saw it again in this case, and I was glad of an opportunity of pointing it out to Mr. Farrant, a man of much observation and respectability, and Surgeon of the First Regiment of Devon Militia. In this instance this areolary character disappeared on the following day. But a small proportion of these eruptions could be said to reach maturation, and all these were fully maturated as early as the fourth and fifth day, indeed some were already desiccating, and on the morning of the sixth most of them were entirely swept away. The few that remained were those which had nearly reached the medium size of small-pox, these were perfectly scabbed, were flattened, and of a dark mahogany tint; in short, were unquestionably more resembling in all their characters small vaccinæ than variolæ. Messrs. Sargent and Little saw them in this stage. Dr. May, and I believe some other medical men, also saw this case; no doubts were entertained by any one that the affection was variolous. I vaccinated this child, Oct. 8, 1803; the cicatrix on one arm is distinctly, if not strongly marked, but I observe no seaminess in it; on the other arm any vestige is with difficulty discovered. Fluid was taken from this child largely two or three days successively.

I inoculated a child in this family about ten years ago, with small-pox; this child had the confluent disease, and died on the tenth or eleventh day of it; and this family had before lost a child in the casual disease: we have here then another instance of what I have ventured to term

*incomplete*

*incomplete vaccination* happening in a family, and in all probability in a subject of high variolous susceptibility, and again followed by a sort of hybrid result or modified variolæ. Such and so often have been these coincidences, they would hardly seem to be accidental; I do not think, therefore, it can justly be deemed gratuitous assumption, to connect and consider them as cause and effect. I have the satisfaction to know that this view of them is at this time fully subscribed by several of the medical men in these towns, who are at the top of the profession. Another child in this family, whom I vaccinated July 7, 1800, has to this day entirely resisted the small-pox. Mrs. Jackman says, she does not recollect that the vesicle on this child's arm was ever broken or opened.

If future observation does not develope other more direct and adequate causes of variolous action subsequent to vaccination, I hope it will tend at least to confirm these and others which have been already adduced. It is undoubtedly a great desideratum, that we should on these occurrences have a something on which the mind can generally rest with tolerable satisfaction; a few cases have occurred, and will of course again occur in both inoculations, which leave us no ground for conjectures with respect to the causes of them. To such inscrutable decisions of Nature we must bow with silence and with respect.

Another case, similar to the former in many of its circumstances, lately fell under our observation at Stone House, in the family of a Mr. Williams, who lives in George's Street; the subject of it is a child whom I vaccinated more than two years ago; nothing could be more regular and correct than the progress of the early vesicles; on the seventh or eighth day the child tore them both to pieces with its nails. From the irregular appearances which followed this destruction of the vesicles, I advertized the parents that I was not satisfied with it as a case of complete vaccination. Mr. and Mrs. Williams often mentioned this remark of mine to their neighbours. The cicatrices on this child's arms are not unusually small, and in many respects are satisfactory. This narrow street had for some time been previously full of the small-pox; some weeks ago, after two or three days of indisposition, somewhere between fifty and a hundred eruptions appeared on this child; these reached their utmost maturation in five days, were very small, and were very suddenly swept away; the areolary character so often attendant on these secondary small-pox was in this case very remarkable, and remained three or four days; indeed, these pustules were more vaccinated than even those in the preceding case; with the event of this case the parents were so much pleased, that they desired me to vaccinate immediately their youngest child. I am sorry however that I did not succeed in infecting it, that it has since suffered severely from the casual disease, and is at this moment suffering from violent ophthalmy.

I am, &c.

*Dock, Aug.* 13, 1805. RICHARD DUNNING.

P. S. The small-pox continues very mortal with us; a few days ago five children were buried on the same evening at Stone House, and some are every day buried. I am at this moment in attendance on a poor babe suffering severely under a confluent disease from an inoculation by the unmedical person who, we believe, has so generally spread this dreadful plague through this town and neighbourhood.

